# Metastatic breast cancer cells inhibit osteoblast differentiation through the Runx2/CBFβ-dependent expression of the Wnt antagonist, sclerostin

**DOI:** 10.1186/bcr3048

**Published:** 2011-10-27

**Authors:** Daniel Mendoza-Villanueva, Leo Zeef, Paul Shore

**Affiliations:** 1 Faculty of Life Sciences, University of Manchester, Michael Smith Building, Oxford Road, Manchester, M13 9PT, UK

## Abstract

**Introduction:**

Breast cancers frequently metastasise to the skeleton where they cause osteolytic bone destruction by stimulating osteoclasts to resorb bone and by preventing osteoblasts from producing new bone. The Runt-related transcription factor 2, Runx2, is an important determinant of bone metastasis in breast cancer. Runx2 is known to mediate activation of osteoclast activity and inhibition of osteoblast differentiation by metastatic breast cancer cells. However, while Runx2-regulated genes that mediate osteoclast activation have been identified, how Runx2 determines inhibition of osteoblasts is unknown.

**Methods:**

The aim of this study was to determine how Runx2 mediates the ability of metastatic breast cancer cells to modulate the activity of bone cells. We have previously demonstrated that Runx2 requires the co-activator core binding factor beta (CBFβ) to regulate gene expression in breast cancer cells. We, therefore, performed independent microarray analyses to identify target genes whose expression is dependent upon both Runx2 and CBFβ. Common target genes, with a role in modulating bone-cell function, were confirmed using a combination of siRNA, quantitative reverse transcriptase PCR (qRT-PCR), ELISA, promoter reporter analysis, Electrophoretic Mobility Shift Assay (EMSA) and chromatin immunoprecipitation (ChIP) assays. The function of Runx2/CBFβ-regulated genes in mediating the ability of MDA-MB-231 to inhibit osteoblast differentiation was subsequently established in primary bone marrow stromal cell cultures and MC-3T3 osteoblast cells.

**Results:**

We show that Runx2/CBFβ mediates inhibition of osteoblast differentiation by MDA-MB-231 cells through induction of the Wnt signaling antagonist, sclerostin. We demonstrate that MDA-MB-231 cells secrete sclerostin and that sclerostin-expression is critically dependent on both Runx2 and CBFβ. We also identified the osteoclast activators IL-11 and granulocyte-macrophage colony-stimulating factor (GM-CSF) as new target genes of Runx2/CBFβ in metastatic breast cancer cells.

**Conclusions:**

This study demonstrates that Runx2 and CBFβ are required for the expression of genes that mediate the ability of metastatic breast cancer cells to directly modulate both osteoclast and osteoblast function. We also show that Runx2-dependent inhibition of osteoblast differentiation by breast cancer cells is mediated through the Wnt antagonist, sclerostin.

## Introduction

Breast cancer bone metastases cause osteolytic bone destruction by interrupting the normal bone remodelling process [1-3]. These metastatic tumours interfere with normal bone remodelling by stimulating osteoclasts to resorb bone and preventing new bone growth by inhibiting osteoblasts. Thus, bone degradation is due to both increased activation of osteoclasts and suppression of osteoblasts. Existing drug therapies primarily target the osteoclasts to prevent bone resorption [4]. However, while this approach reduces the bone lesions and improves the quality of patient life, therapies that restore osteoblast activity are required to achieve more complete repair of osteolytic lesions [1]. It is well established that breast cancer cells induce osteolytic lesions by secreting soluble factors that lead to osteoclast-mediated bone resorption. Such factors include osteopontin, parathyroid hormone-related protein (PTHrP), IL-11, IL-8, receptor activator of nuclear factor kappa-B ligand (RANKL) and GM-CSF, all of which stimulate osteoclasts either directly or indirectly [3]. In contrast, few studies have identified mechanisms by which breast cancer cells suppress bone synthesis by osteoblasts. Breast cancer cells secrete Dickkopf-related protein 1(DKK1), an antagonist of the Wnt/β-catenin pathway that blocks osteoblast differentiation [5]. Members of the transforming growth factor beta (TGFβ) family also contribute to inhibition of osteoblast differentiation by metastatic breast cancer cells [6,7].

The Runt-related transcription factor 2, Runx2, is a master-regulator of bone development and an important determinant of bone metastasis in breast cancer cells [8-10]. In breast cancers, Runx2 is aberrantly expressed and inhibition of Runx2 function in metastatic breast cancer cells transplanted to bone prevents the formation of osteolytic lesions [8]. Runx2 contains a conserved DNA-binding domain, termed the Runt domain, that recognises the consensus sequence ACC(A/G)CA [11]. The Runt domain also interacts with its co-activator core binding factor beta (CBFβ). CBFβ stimulates DNA-binding of the Runt domain, and is essential for most of the known functions of Runx2, including activation of Runx2-regulated genes in breast cancer cells [12,13].

Runx2 contributes to the ability of breast cancer cells to activate osteoclasts by up-regulating the expression of Indian Hedgehog (IHH) [14]. IHH subsequently stimulates PTHrP-induced RANKL production by osteoblasts, which in turn activates osteoclasts [15,16]. Runx2 also regulates expression of osteopontin (OPN), which binds to the CD44 receptor to activate osteoclasts [15,16]. Previous reports showed that Runx2 expression in metastatic breast cancer cells, and in prostate cancer cells, also contributes to their ability to inhibit osteoblast differentiation [8,17]. However, the molecular mechanism by which Runx2 mediates osteoblast inhibition remains unknown. The aim of this study was to identify genes regulated by Runx2/CBFβ that contribute to the ability of metastatic breast cancer cells to modulate the activity of bone cells. Since we had previously established that CBFβ contributed to the function of Runx2 in MDA-MB-231 cells we reasoned that independent microarray analyses would identify the Runx2/CBFβ-regulated genes [18]. This analysis identified the osteoclastogenic cytokines IL-11 and granulocyte-macrophage colony-stimulating factor (GM-CSF) as new Runx2/CBFβ targets in breast cancer cells. We also show that Runx2/CBFβ regulates expression of the Wnt antagonist sclerostin and thus reveal a mechanism by which Runx2 mediates osteoblast inhibition by breast cancer cells.

## Materials and methods

### Cells and culture conditions

Human non-metastatic MCF7 cells and metastatic MDA-MB-231 cells were obtained from ATCC and cultured as previously described [18]. Mouse pre-osteoblastic cells (MC3T3-E1) were obtained from ATCC and cultured in α-MEM plus 10% FBS and 1% P/S. For differentiation studies, MC3T3-E1 cells were cultured in differentiation media containing 10 mM β-glycerol phosphate (Sigma, St. Louis, MO, USA) and 50 μg/ml L-ascorbic acid (Sigma). Conditioned medium from MDA-MB-231 cells was collected as previously described [6]. Depletion of sclerostin from the conditioned media was carried out incubating the media with 4 μg of the sclerostin (Ab63097, Abcam, Cambridge, UK) or Flag antibody (F1804, Sigma) per ml of media followed by incubation with Protein G agarose beads. Centrifuged beads were removed and the media was used for the experiments. MC3T3-E1 cells were cultured with conditioned medium or sclerostin-free conditioned media according to reported protocol [6]. Cells were differentiated for 18 days and then used either for alizarin red staining or total mRNA extraction. Alizarin was quantified from plates after elution following acetic acid protocol as previously reported [19]. Absorbance was measured at 405 nm and relative absorbance was obtained (Abs/Abs Non-diff). One-way analysis of variance was used to determine statistical differences between groups using SPSS software (Chicago, IL, USA). A *P-*value < 0.05 was considered statistically significant. Multiple comparisons between individual groups were assessed by the method of Tukey. Mouse bone marrow stromal cells (BMSCs) were extracted as previously described [20]. Mice were bred in a designated establishment, in accordance with the Animals (Scientific Procedures) Act 1986, at the University of Manchester (designation no. 5/2560). Mice were euthanized prior to harvesting of BMSCs. These procedures do not require Institutional Ethics Board approval since they fall outside the Animals (Scientific Procedures) Act 1986. Cells were then seeded into six-well plates, 5.6 × 10^6 ^cells/well (Day 0). After 48 hours the growth medium was changed to differentiation media and treated as the MC3T3-E1 cells.

### Microarrays

For each microarray analysis, MDA-MB-231 cells were transfected with siRNAs. In total, three independent replicates treated with siRNA against Runx2, CBFβ or a non-specific (siNS) were employed. Total RNA was extracted using RNeasy Mini Kit (Qiagen GmbH, Hilden, Germany) according to the manufacturer's instructions. The quality and size distribution of the RNA were assessed with the RNA Nano Labchip kit (Agilent Technologies, Waldbronn, Germany). Samples were hybridised with the Human Genome U133 Plus 2.0 Array, according to the manufacturer's instructions (Affymetrix, Santa Clara, CA, USA). Each microarray was performed in triplicate for statistical robustness. Technical quality control was performed with dChip (V2005) using the default settings [21,22]. Background correction, quantile normalisation and gene expression analysis were performed using RMA in Bioconductor [23]. Principal component analysis (PCA) was performed with Partek Genomics Solution (version 6.5, Copyright 2010, Partek Inc., St. Charles, MO, USA). Differential expression analysis was performed using Limma using the functions lmFit and eBayes [24]. Gene lists of differentially expressed genes were controlled for false discovery rate (fdr) errors using the method of QVALUE [25]. The microarray data were deposited via ArrayExpress with the accession number: E-MEXP-3230.

### Transient transfection and siRNAs

For the MCF-7 transfections of Runx2, the plasmid pRK-Flag-Cbfa1 containing the wild-type Runx2 protein or the parent plasmid was used [9]. Transfections were performed using lipofectamine 2000 (Invitrogen, Carlsbad, CA, USA). MDA-MB-231 cells transfected with human siRNA against CBFβ (siCBFβ), Runx2 (siRunx2), or a non-specific siRNA (siNS) (Santa Cruz Biotechnology Inc., Santa Cruz, CA, USA), were performed with oligofectamine (Invitrogen).

### Generation of stable MDA-MB-231 cell lines

Cell lines were established using short hairpin (sh) RNA lentiviral particles for human CBFβ, Runx2 or control with a non specific shRNA according to the suppliers instructions (Santa Cruz Biotechnology, Inc.). Stable cell lines expressing the shRNA were selected by the addition of 1 μg/ml puromycin. Clones were assessed for the expression of CBFβ or Runx2 by Western blot.

### Luciferase assays

The human *SOST *promoter (SOST WT) in pGL3B plasmid encoding the wild-type promoter sequence (2 kb upstream of the 5'-UTR) has been described previously [26]. The mutant *SOST *promoter reporter (SOST Mut) was generated by site directed mutagenesis (Stratagene, La Jolla, CA, USA), changing the three consensus Runx2-binding sites, ACCACA, to ATGACA. The human GM-CSF promoter/enhancer (CSF-2 WT) reporter plasmid and the plasmid with the Runx2 sites mutated in the promoter and the enhancer (CSF-2 Mut) has been described previously [27]. Luciferase assays were performed as described previously [9].

### ChIP assays

ChIP assays were performed as described previously [18]. Real time PCR was performed with *SOST *promoter primers (forward: 5'-AAGCCGGAGCTCATTTTGATA-3'; reverse: 5'-CCTCCCCAAAGACTTCTCCTC-3'); *CSF-2 *promoter primers (forward: 5'-GTTCCCATGTGTGGCTGATAAG-3' reverse: 5'-TCTGTGTACTGGGCTCACTGG-3') and 18S gene primers (forward: 5'-GTAACCCGTTGAACCCCATT-3' reverse: 5'-CCATCCAATCGGTAGTAGCG-3').

### ELISA assays

MDA-MB-231 cells were transfected with siRunx2 or siNS and after 48 h ELISA were performed to measure the amount of sclerostin and GM-CSF in the media. Immuno-detection was performed using ELISA assay kits BI-20492 (Biomedica Gruppe, Vienna, Austria) and DGM00 (R&D Systems, Minneapolis, MN, USA) respectively. Data are presented as mean ± standard deviation (SD).

### RT-PCR

Total mRNA was isolated from cell pellets using an RNeasy mini kit (Qiagen); genomic DNA was removed using the RNase-free DNase set (Qiagen). mRNA expression of the selected genes was determined by real-time PCR using one-step QuantiTect SYBR Green RT-PCR kit (Qiagen) according to the manufacturer's protocol. Nucleotide sequences of specific primers for the different human genes can be supplied on request. Data were analysed as described previously [18]. For comparison of mRNA levels, the data were analyzed using the equation described by Livak [28].

### Immunofluorescence

Cells grown on coverslips were fixed with 4% paraformaldehyde, permeabilised with 0.1% Triton X-100, and blocked with 1% bovine serum albumin in phosphate-buffered saline. Signals were detected by an indirect immunofluorescence technique using primary antibodies with a mouse monoclonal anti-Runx2 (D130-3, MBL International) or rabbit polyclonal anti-CBFβ antibodies (Ab33516, Abcam) and Alexa fluor secondary antibodies (A11005 and A11008, Invitrogen). Images were processed using MacBiophotonics ImageJ (Bethesda, Maryland, USA).

### Western blot

Proteins were detected with a mouse monoclonal anti-Runx2 (MBL International) or rabbit polyclonal anti-CBFβ antibody (Abcam). Goat anti-mouse IgG conjugated with horseradish peroxidase (Pierce, Rockford, IL, USA), was used as a secondary antibody. Immune complexes were detected by Supersignal West Dura Extended Duration Substrate according to the manufacturers' protocol (Pierce).

### Electrophoretic Mobility Shift Assay (EMSA)

EMSAs were performed as described previously [9]. The DNA sequence of the oligonucleotides used were as follows: SOST, sense 5'CAGTTCTGAAAACCACAGCCGCCA3', antisense 5'CAGTTGGCGGCTGTGGTTTTCAGA3'; *CSF-2*, sense 5'CAGTGGCATTTTGTGGTCACCATT3', antisense 5'CAGTAATGGTGACCACAAAATGCC3'; SOST Mut, sense 5'CAGTTCTGAAACAGTCAGCCGCCA3', antisense 5'CAGTTGGCGGCTGACTGTTTCAGA3'; *CSF-2 *Mut, sense 5'CAGTGGCATTTATTGTGCATCATT3', antisense 5'CAGTAATGATGCACAATAAATGCC3'.

## Results

### Runx2 and CBFβ in metastatic breast cancer cells regulate activators of osteoclasts and inhibitors of osteoblasts

**Figure 1:**
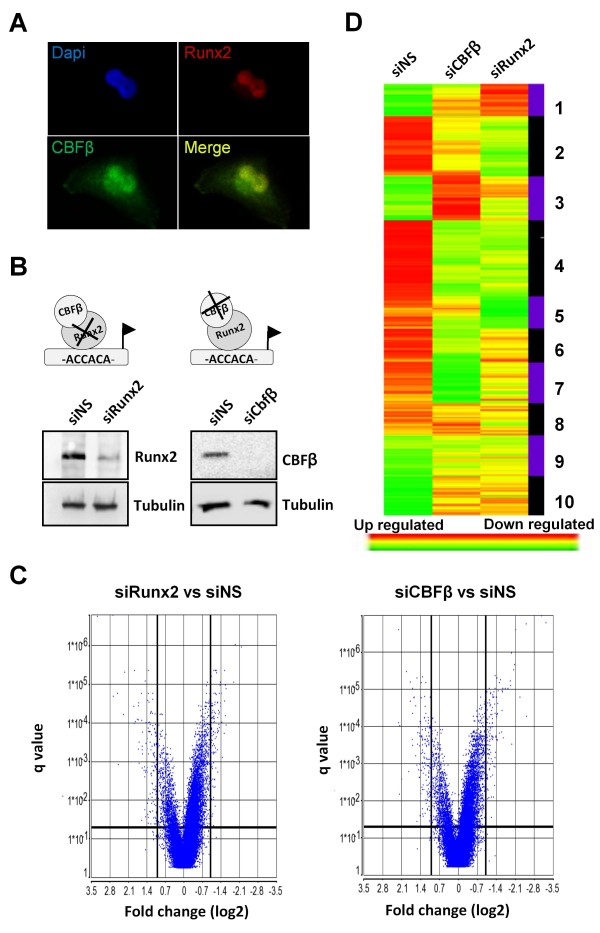
**Independent microarray analyses identify new targets of Runx2 and CBFβ in metastatic MDA-MB-231 cells**. **(A) **Fluorescence microscopy showing co-localization of Runx2 and CBFβ in the nucleus of MDA-MB-231 cells. MDA-MB-231 cells were immunostained with specific antibodies as indicated. Nuclei (blue) were stained with DAPI. **(B) **Western blot showing siRNA knockdown of Runx2 and CBFβ. The upper panel shows total cell lysates derived from siRNA transfections of MDA-MB-231 cells after immunodetection with anti-Runx2 or anti-CBFβ antibodies. The lower panel is a Tubulin loading control. **(C) **Volcano plots of differentially expressed genes in MDA-MB-231 cells treated with siRNAs against Runx2 (siRunx2) or CBFβ (siCBFβ) vs non-specific siRNA control (siNS). The q values were plotted against fold suppression/induction. The darker vertical line indicates a cut off of ≥ 2-fold change and the darker horizontal line represents a q value ≤ 0.05. Each point represents an individual transcript. **(D) **Hierarchical clustering analysis. The colour bar indicates the relative abundance of each transcript with red being the most abundant.

Given that Runx2 is required for the formation of osteolytic metastases we sought to identify target genes that mediate this process. The breast cancer cell line, MDA-MB-231, forms osteolytic bone lesions in mice, and Runx2 is required for this process [8,27]. We have previously shown that Runx2 requires its co-activator, CBFβ, to regulate gene expression in MDA-MB-231 cells [18]. These proteins also co-localise in the nucleus of MDA-MB-231 cells (Figure 1A). To identify Runx2/CBFβ target genes that might be involved in bone metastases, we performed independent microarray analyses using mRNA derived from MDA-MB-231 cells transfected with either siRunx2 or siCBFβ (Figure 1B). Cells transfected with non-specific siRNA (siNS) were used as controls. Transfection of Runx2 siRNAs reduced Runx2 expression by approximately 80% compared to siRNA controls, whereas CBFβ was undetectable in cells transfected with CBFβ siRNA (Figure 1B). Three independent biological samples for each condition were used for array hybridizations. Hybridizations were performed for each of the samples using the Human Genome U133 Plus 2.0 Array. Of the 38,500 genes on the microarrays, we identified 357 genes in the siRunx2-transfected cells and 340 in the siCBFβ-transfected cells, whose expression changed by ≥ 2-fold. The data are presented as a volcano plot (Figure 1C). Genes whose expression changed significantly by ≥ 2-fold (*q*-value < 0.05) are located in the upper-left and upper-right quadrants of the plot. Hierarchical clustering analysis of these genes was employed to characterize the expression patterns in the three conditions (Figure 1D). Distinct patterns of expression differences were observed for Runx2 and CBFβ samples compared to NS.

**Figure 2:**
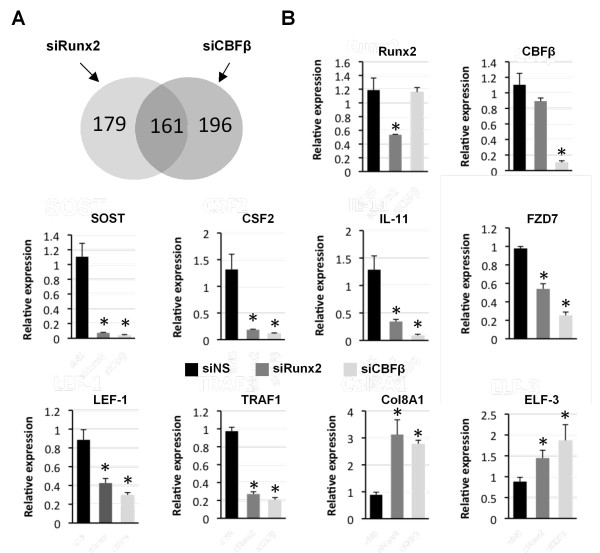
**Runx2 and CBFβ are required for the expression of genes encoding the soluble ligands, IL-11, sclerostin and GM-CSF in MDA-MB-231 cells**. **(A) **Venn diagram showing the number of genes with ≥ 2-fold expression-change in each microarray. **(B) **Validation of Runx2 and CBFβ target genes. MDA-MB-231 cells transfected with siRunx2, siCBFβ or a non-specific siRNA were subject to real-time qRT-PCR. Acidic ribosomal phosphoprotein P0 (RPLO) mRNA was used as a control for normalization and relative values are shown. Data are presented as mean ± standard deviation (SD) (*n *= 3). Statistical evaluation of significant differences was performed using the Student's t-test. Asterisk (*) indicates *P *< 0.05 when compared to control (siNS).

A comparison of genes whose expression changed in the Runx2 and CBFβ knockdown cells revealed 161 common genes (Figure 2A; Additional file 1). A change in expression of a selection of these genes was subsequently verified using qRT-PCR. All genes tested by qRT-PCR displayed significant changes in expression in both the Runx2 and CBFβ knockdown cells (Figure 2B). No significant reduction in Runx2 expression was observed in siCBFβ-transfected cells, nor was there a significant reduction in CBFβ expression in siRunx2-transfected cells, indicating that changes in expression are not due to downstream reciprocal changes in Runx2 or CBFβ expression. From the 161 common genes, 86 were down-regulated and, therefore, are potentially activated by Runx2/CBFβ. These genes were subjected to gene ontology analysis using the Database for Annotation, Visualization and Integrated Discovery (DAVID) analysis online tool (Additional file 2 Table S1) [29]. This analysis identifed a number of genes that encode secreted proteins. Within this group we identifed three genes encoding soluble ligands with established roles in bone-related processes, *IL-11*, *CSF-2 *and *SOST*. IL-11 and GM-CSF (encoded by *CSF-2*) are osteoclastogenic cytokines whose expression by metastatic breast cancer cells is known to stimulate bone resorption by osteoclasts [30,31]. In contrast, *SOST *encodes an inhibitor of bone formation, the Wnt antagonist sclerostin [32]. Sclerostin is normally secreted by osteocytes to inhibit osteoblast differentiation. However, it has not previously been implicated in breast cancer bone metastasis.

### Runx2 directly regulates expression of *SOST *and *CSF-2 *genes in MDA-MB-231 cells

**Figure 3:**
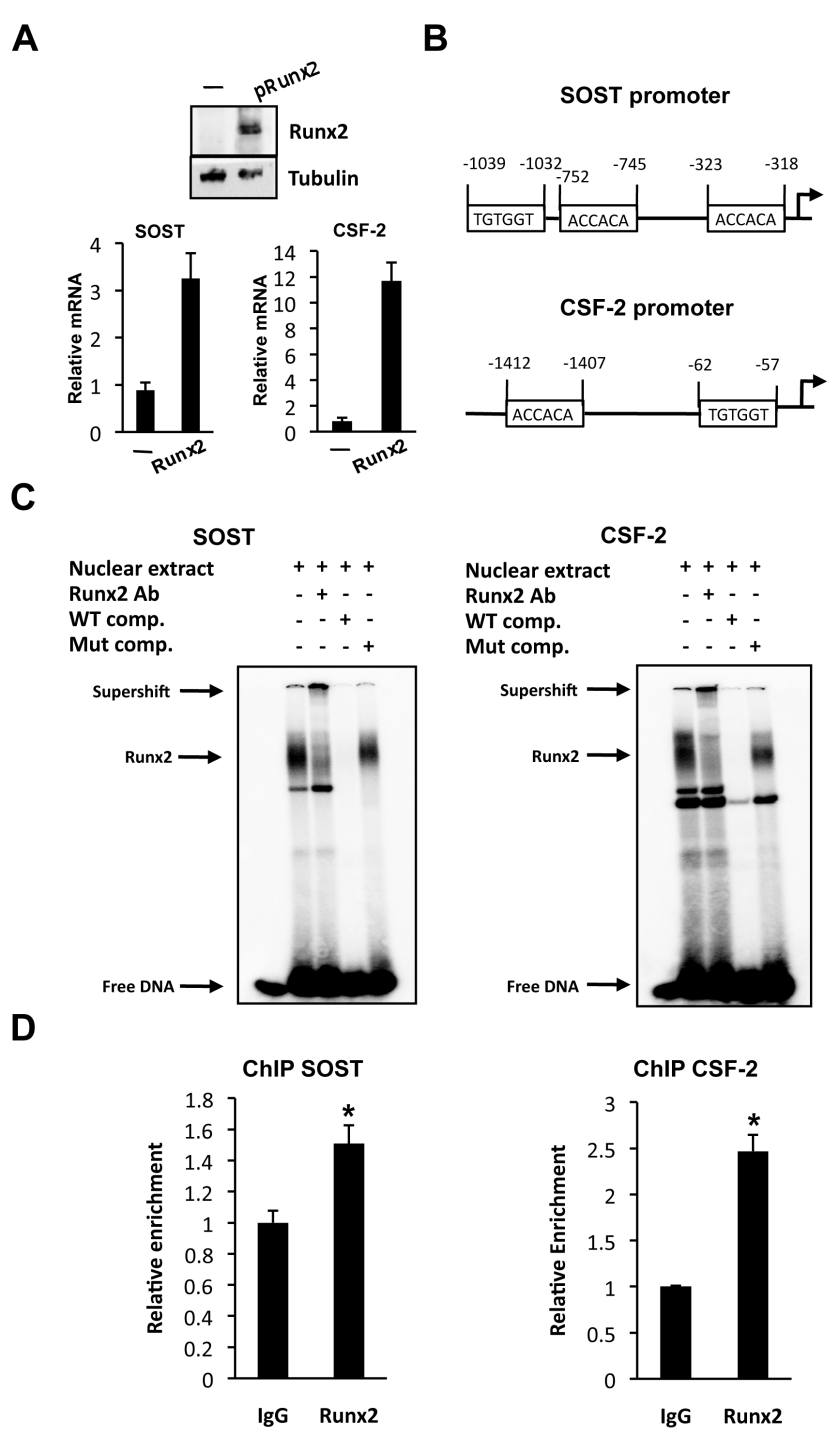
**Runx2 binds to the *SOST *and *CSF-2 *promoter**. **(A) **Western blot showing Runx2 expression after transfection in non-metastatic MCF-7 cells. MCF-7 cells were transfected with a Runx2-expression plasmid or the parent plasmid (-). The upper panel shows total cell lysates derived from transfections of MCF-7 cells after immunodetection with an anti-Runx2 antibody. The lower panel is a Tubulin loading control. The graphs show an increase in *SOST *and *CSF-2 *expression in non-metastatic MCF-7 cells after transfection with a Runx2-expression plasmid. **(B) **Diagram showing the localization of the Runx2 binding sites in the human *SOST *and *CSF-2 *promoter. The arrow indicates the start of transcription. **(C) **EMSA demonstrating specific binding of Runx2 to the Runx elements in the *SOST *and *CSF-2 *promoters. Nuclear extracts from MDA-MB-231 were incubated with Runx-binding sites from the human *SOST *or *CSF-2 *gene promoters. Competition assays were performed with a 100-fold molar excess of either cold wild-type (WT comp.) or mutant (Mut comp.) probes. Each gel is representative of three experiments. **(D) **Runx2 is recruited to the *SOST *and *CSF-2 *promoter in MDA-MB-231 cells. ChIP assays using Runx2 antibodies. Data are presented as mean ± standard deviation (S.D.) (*n *= 3). * indicates *P *< 0.05 compared with IgG by analysis of variance.

Previous studies have shown that Runx factors directly regulate *SOST *and *CSF-2 *expression in other cell-types but they are not known targets of Runx2 in breast cancer cells [27,33]. We, therefore, focussed our analysis on the regulation of *SOST *and *CSF-2 *by Runx2 in MDA-MB-231 cells. We first demonstrated that expression of *SOST *and *CSF-2 *could be induced in non-metastatic MCF-7 breast cancer cells by heterologous expression of Runx2 (Figure 3A). MCF-7 cells express CBFβ but not Runx2. Transfection of MCF-7 cells with a Runx2-expression plasmid resulted in a significant increase in *SOST *and *CSF-2 *mRNA, thus confirming that aberrant expression of Runx2 can drive *SOST *and *CSF-2 *expression (Figure 3A). We also demonstrated that endogenous Runx2 in MDA-MB-231 cells bind to the Runx sites in both gene promoters (Figure 3B). Nuclear extracts from MDA-MB-231 cells were used in EMSAs to demonstrate specific binding of Runx2 to the promoter regions (Figure 3C). Incubation of radiolabelled Runx2-binding sites from *SOST *and *CSF-2 *promoters resulted in specific complex formation that could be ablated by specific competitor DNA but not by competitor DNA in which the Runx site was mutated. The complex was also supershifted by the addition of a Runx2 antibody. We also performed ChIP experiments on chromatin derived from MDA-MB-231 cells (Figure 3D). Both the *SOST *and *CSF-2 *promoters were enriched in chromatin precipitated with Runx2 antibody as compared to a non-specific control IgG antibody.

**Figure 4:**
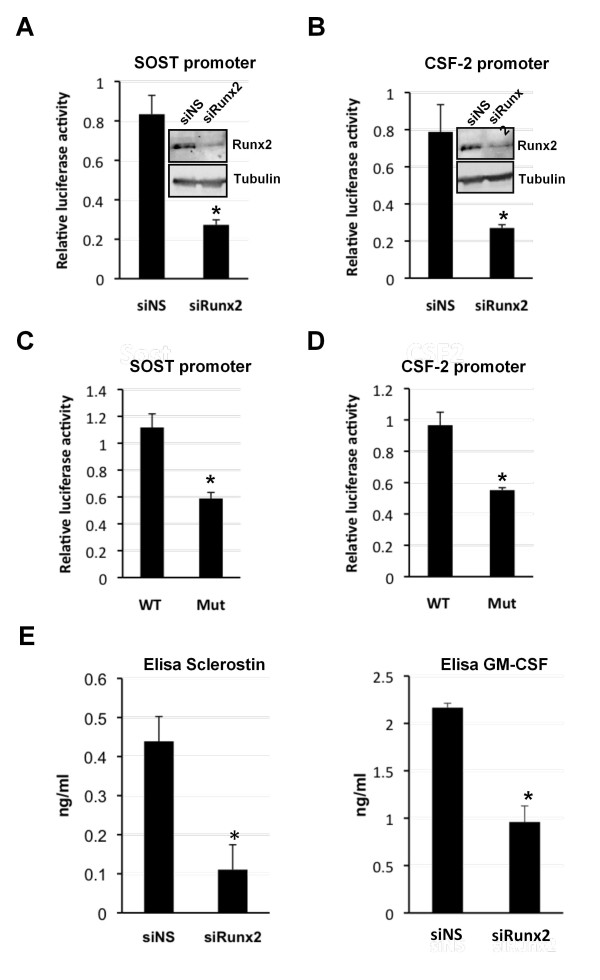
**Runx2 is essential for the expression of secreted sclerostin and GM-CSF in MDA-MB-231 cells**. **(A and B) **Runx2 regulates the activity of the *SOST *and *CSF-2 *promoter. MDA-MB-231 cells transfected with siRunx2 or a non-specific siRNA (siNS) were transfected with a plasmid containing either the *SOST *(A) or *CSF-2 *(B) promoter. Luciferase activities are presented as mean values ± SD All values are relative to the activity of the Renilla luciferase reporter. Insets show Western blots of Runx2 expression when transfected with siRNAs as indicated. **(C and D) **MDA-MB-231 cells transfected with the luciferase reporter constructs containing, either the *SOST *(C) or *CSF-2 *(D) wild-type promoter (WT), or the Runx site mutant (Mut). Data are presented as mean ± standard deviation (SD) (*n *= 3). All values are relative to the activity of the Renilla luciferase reporter. **(E) **ELISA showing sclerostin and GM-CSF in the media of MDA-MB-231 cells. Data are presented as mean ± standard deviation (SD) (*n *= 3). Statistical evaluation of significant differences was performed on all graphical data using the Student's t-test. Asterisk (*) indicates *P *< 0.05 when compared to controls.

To determine if Runx2 directly activates the *SOST *and *CSF-2 *promoters, MDA-MB-231 cells were transfected with siRunx2 or non-specific siRNA (siNS) followed by transfection with a luciferase reporter plasmid containing the human *SOST *or *CSF-2 *promoters (Figure 4). In the Runx2-knockdown cells the activity of both promoters was significantly decreased compared to control cells (Figure 4A, B). Moreover, mutation of the Runx2-binding sites in either of the promoters resulted in reduced promoter activity (Figure 4C, D).

To examine the effect of knocking down Runx2 expression in MDA-MB-231 cells on protein expression ELISA was used to measure the amount of sclerostin and GM-CSF secreted into the media (Figure 4E). Cells transfected with siRunx2 produced approximately five-fold less sclerostin than control cells. GM-CSF was reduced by almost three-fold. Taken together these data demonstrate that Runx2 is required for the production of sclerostin and GM-CSF by activating expression of the *SOST *and *CSF-2 *genes in MDA-MB-231 cells. Thus, Runx2 regulates the expression of soluble protein ligands that either stimulate osteoclast activity or inhibit osteoblast differentiation.

### Sclerostin mediates inhibition of osteoblast differentiation by MDA-MB-231 cells

While the role of GM-CSF as an activator of osteoclasts in breast cancer bone metastasis is well established, the role of sclerostin has not been investigated [28]. Indeed, very little is known about the mechanisms by which metastatic breast cancer cells inhibit osteoblast differentiation. Moreover, how Runx2 mediates osteoblast inhibition has not been investigated. Our finding that Runx2/CBFβ drives *SOST *expression in MDA-MB-231 suggested that sclerostin secretion by MDA-MB-231 cells might determine their ability to inhibit osteoblast differentiation.

**Figure 5:**
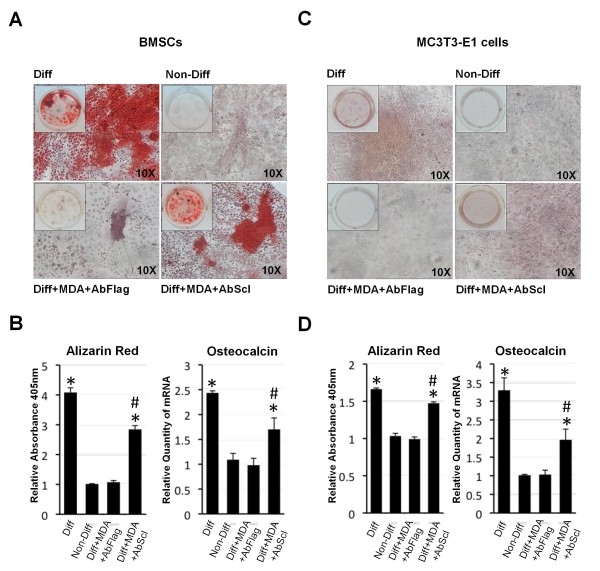
**Sclerostin mediates inhibition of osteoblast differentiation by MDA-MB-231 cells**. **(A) **Depletion of sclerostin from MDA-MB-231-conditioned media permits osteoblast differentiation in primary bone marrow stromal cell cultures (BMSCs). BMSCs were differentiated with normal differentiation media (Diff) or MDA-MB-231-conditioned media (MDA). Cells grown in non-differentiation media (Non-Diff) were used as negative control. MDA-MB-231 media were depleted by immunoprecipitation with a sclerostin antibody (AbScl) or a control Flag antibody (AbFlag) as indicated below the panels. **(B) **Alizarin red was quantified following elution and total RNAs from cells treated as in (A) were used to analyse expression of the osteoblast differentiation marker, osteocalcin. Data are presented as mean ± standard deviation (SD) (*n *= 3). One-way analysis of variance was used and multiple comparisons between individual groups were assessed by the method of Tukey. * indicates *P *< 0.05 compared to Non-Diff by analysis of variance; # indicates *P *< 0.05 compared to Diff+MDA+AbFlag by analysis of variance. **(C) **Sclerostin inhibits the differentiation of the pre-osteoblastic cell line MC3T3-E1. MC3T3-E1 cells were treated as in (A). **(D) **Alizarin red and mRNA levels of osteocalcin were determined. Data were analysed as in (B).

Previous studies have shown that MDA-MB-231-conditioned media inhibits the differentiation of osteoblast precursors in bone marrow stromal cell cultures (BMSCs) [34]. To determine if sclerostin expression by MDA-MB-231 cells contributes to their ability to inhibit differentiation of osteoblasts, we, therefore, examined the effect of sclerostin-depleted MDA-MB-231-conditioned media on osteoblast differentiation in BMSCs (Figure 5A). Differentiation of BMSCs was induced by the addition of ascorbic acid and β-glycerol phosphate and assessed by the presence of calcium deposition with alizarin red after 18 days. Alizarin red was quantified by measuring absorbance at 405 nm after elution with acetic acid (Figure 5B) [19]. Sclerostin was depleted in MDA-MB-231-conditioned media by immunoprecipitation with a sclerostin-specific antibody prior to addition to the BMSC culture. Media treated with an anti-Flag antibody was used in controls. As expected, in non-conditioned differentiation media, significant alizarin red staining was observed in BMSC cultures after 18 days and no staining was observed in cultures grown in non-differentiation media (Figure 5A, B). Also as expected, differentiation of BMSCs was significantly inhibited when grown in conditioned differentiation media derived from MDA-MB-231 cells (Figure 5A, B). The anti-Flag treated media also inhibited differentiation of the BMSCs as observed with MDA-MB-231 conditioned media (Figure 5A, B). In contrast, significant differentiation was observed in BMSCs cultured in sclerostin-depleted media (Figure 5A, B). In addition, qRT-PCR demonstrated that expression of the osteoblast differentiation marker, osteocalcin, was increased in BMSCs grown in sclerostin-depleted media, but not in cells grown in the anti-Flag treated media (Figure 5B). We also performed the same experiments on the pre-osteoblast cell line MC3T3-E1, which can also be induced to differentiate to mature calcium-depositing osteoblasts (Figure 5C, D) [6]. As with the BMSC cultures, anti-Flag treated media inhibited differentiation and osteocalcin expression, whereas cells treated with sclerostin-depleted media differentiated and osteocalcin expression increased (Figure 5C, D). Thus, inhibition of osteoblast differentiation by MDA-MB-231 is mediated by sclerostin.

### Runx2 expression in MDA-MB-231 cells is required for inhibition of osteoblast differentiation

**Figure 6:**
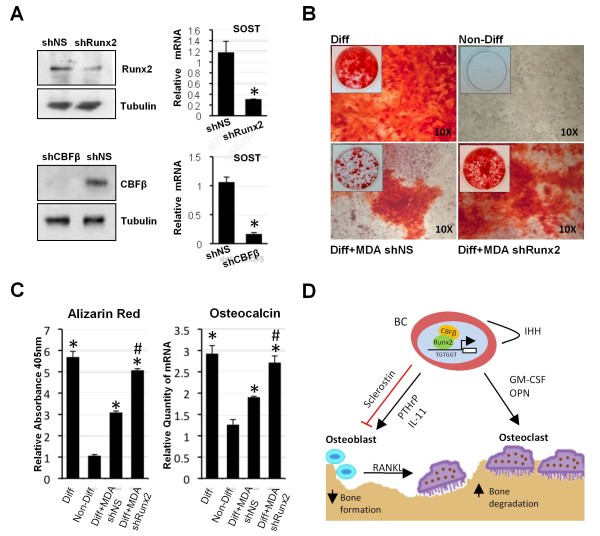
**Runx2 expression in MDA-MB-231 cells is required for inhibition of osteoblast differentiation**. **(A) **Western blot showing stable MDA-MB-231 cells knocked down for Runx2 or CBFβ. The left panel shows total cell lysates derived from stables MDA-MB-231 cells, transfected with a shRNA non-specific (shNS) or shRNA for Runx2 (shRunx2) or CBFβ (shCBFβ). The lower panel is a Tubulin loading control. Graphs show that expression *SOST *is significantly reduced in the cell lines. Data are presented as mean ± standard deviation (SD) (*n *= 3). Statistical evaluation of significant differences was performed using the Student's t-test. Asterisk (*) indicates *P *< 0.05 when compared to control (shNS). **(B) **Knock-down of Runx2 in MDA-MB-231 cells permits the differentiation of osteoblasts. BMSCs were differentiated with normal differentiation media (Diff), conditioned media from stable shNS cells (MDAshNS) or shRunx2 cells (MDAshRunx2). Cells grown in non-differentiation media (Non-diff) were used as negative control of differentiation. **(C) **Alizarin red was quantified following elution and total RNAs from cells treated as in (B) were used to analyse expression of the osteoblast differentiation marker, osteocalcin. Data are presented as mean ± standard deviation (SD) (*n *= 3). One-way analysis of variance was used and multiple comparisons between individual groups were assessed by the method of Tukey. * indicates *P *< 0.05 compared to Non-Diff by analysis of variance; # indicates *P *< 0.05 compared to Diff+MDA shNS by analysis of variance. **(D) **Diagram depicting the proposed role of Runx2 in the modulation of bone cell functions by regulating secreted factors. Other genes involved in invasion are omitted for clarity.

A previous study demonstrated that expression of a dominant-negative form of Runx2 in MDA-MB-231 prevented inhibition of osteoblasts in co-culture with BMSCs [8]. We predicted that knockdown of Runx2 expression in MDA-MD-231 cells should permit osteoblast differentiation by MDA-MB-231-conditioned media. We, therefore, examined the effect of knocking down the expression of Runx2 on the ability of MDA-MB-231-conditioned media to suppress osteoblast differentiation. We generated stably transfected MDA-MB-231 cells, in which either Runx2 (MDA-shRunx2 cells) or CBFβ (MDA-shCBFβ cells) was knocked down by shRNAs (Figure 6). In both cell lines sclerostin expression was significantly reduced compared to control cells harbouring non-specific shRNA (MDA-shNS cells) (Figure 6A). Thus, both transient and stable RNAi treatments, independently targeting either Runx2 or CBFβ, resulted in down-regulation of sclerostin expression. Taken together with the induction of sclerostin mRNA by exogenous Runx2 in MCF-7 cells, it is unlikely that the RNAi effects on sclerostin expression are due to off-target effects of the RNAi. We conclude that sclerostin expression is dependent on Runx2 and CBFβ.

We next compared the effect of conditioned media derived from MDA-shRunx2 cultures and control MDA-shNS cultures on osteoblast differentiation in BMSCs (Figure 6B, C). Differentiation was induced by the addition of ascorbic acid and β-glycerol phosphate and assessed by the presence of calcium deposition with alizarin red after 18 days. Differentiation of BMSCs was significantly inhibited when grown in conditioned differentiation media derived from MDA-MB-231 cells stably transfected with non-specific shRNA (MDA-shNS) (Figure 6B, C). In contrast, significant alizarin red staining was observed in BMSC cultures grown in conditioned differentiation media derived from MDA-shRunx2 cells (Figure 6B, C). Osteocalcin expression was also increased in BMSCs grown in differentiation media derived from MDA-shRunx2 cells compared to BMSCs grown in differentiation media derived from non-specific shRNA (MDA-shNS) (Figure 6C). Taken together, our data show that Runx2 mediates inhibition of osteoblast differentiation through induction of sclerostin secretion by MDA-MB-231 cells.

## Discussion

The aim of this study was to identify Runx2/CBFβ-regulated genes that contribute to the ability of metastatic breast cancer cells to modulate the activity of bone cells. To this end we established that Runx2/CBFβ regulates the expression of genes that mediate both the activation of osteoclasts and the inhibition of osteoblasts by metastatic breast cancer cells (Figure 6C).

While many of the genes we identified have roles in a range of cellular processes that might be important in breast cancers, we focused on genes that have a role in directly modulating bone-cell function. The secreted ligands *IL-11*, *CSF-2 *and *SOST *were identified as Runx2/CBFβ-regulated genes in MDA-MB-231 cells. IL-11 is a potent activator of osteoclast bone resorption [30]. Our finding that Runx2/CBFβ regulates IL-11 is supported by a previous report, which showed that Runx2 up-regulated IL-11 expression in chondrocytes [35]. However, we were unable to identify any consensus Runx-binding sites within the immediate vicinity of the IL-11 promoter. We are presently exploring possible mechanisms by which Runx2/CBFβ regulates IL-11 expression. Runx-binding sites are present within the *CSF-2 *and *SOST *promoters and we demonstrated that recruitment of Runx2 to these sites is necessary for transcription [27,33]. *CSF-2 *is a known target gene of Runx1 in haematopoietic cells [27]. *CSF-2 *encodes the cytokine GM-CSF, which has previously been shown to contribute to osteolytic bone metastasis of breast cancer by stimulating osteoclasts [31]. Thus, our finding that GM-CSF expression is regulated by Runx2 in MDA-MB-231 cells suggests that Runx2 contributes to the formation of osteolytic lesions in metastatic breast cancers, at least in part, by driving the expression of GM-CSF. Previous work has shown that Runx2 contributes to the stimulation of osteoclasts by activating expression of IHH [14]. Runx2 also regulates expression of OPN, which activates osteoclasts [15,16]. We propose that in addition to IHH and OPN, Runx2 mediates stimulation of osteoclast activity by up-regulating the expression of the osteoclastogenic cytokines IL-11 and GM-CSF in metastatic breast cancer cells (Figure 6C).

The most significant finding presented in this study is our demonstration that Runx2 regulates sclerostin expression to inhibit osteoblast differentiation. Sclerostin inhibits osteoblastogenesis by antagonising Wnt signaling [32,36]. In humans, loss-of-function mutations in *SOST *cause sclerosteosis, a disorder in which excessive bone growth occurs. The bones of these patients are also more resistant to fracture. Sclerostin is ordinarily secreted by osteocytes and binds to the co-receptors LRP5 and LRP6 on the surface of osteoblasts to prevent ligand-mediated Wnt signaling, and thus inhibit osteoblast differentiation. Inhibition of osteoblast differentiation is a feature of breast cancer tumour-induced osteolysis. To-date only two factors have been implicated in this process, TGF-β and the Wnt antagonist, DKK1 [5,6]. TGF-β has a number of effects on bone cells, one of which is the inhibition of osteoblast differentiation. DKK1 is a secreted Wnt antagonist that inhibits osteoblast differentiation in a similar way to sclerostin. It has recently been shown that metastatic breast cancer cells secrete DKK1 and that it contributes to their ability to inhibit osteoblast differentiation *in vitro *[5]. There is also evidence that its inhibitory effect on osteoblasts promotes osteolytic metastases in multiple myeloma-associated bone disease [37,38]. Thus, inhibition of osteoblasts by Wnt signalling inhibitors can contribute to the development of osteolytic bone lesions. It is, therefore, likely that both sclerostin and DKK1 cooperate to inhibit Wnt signaling in osteolytic lesions in breast cancer bone metastases. Further studies are, therefore, required to determine if sclerostin contributes to the development of bone metastases *in vivo*.

Existing therapies for bone metastases primarily target the osteoclasts to prevent bone resorption. However, complete healing of bone lesions is not achieved [1,39]. This is likely due, at least in part, to the inhibitory effect of the metastatic tumour cells on osteoblast differentiation and thus new bone synthesis. Therapies that restore osteoblast activity are urgently needed to improve lesion repair. Inhibiting sclerostin, secreted by bone metastases, may prevent inhibition of osteoblast differentiation in osteolytic lesions and thus improve healing. Inhibitors of sclerostin are presently being developed to treat osteoporosis and fractures to increase bone density and aid healing [40,41]. Our finding that metastatic breast cancer cells express sclerostin, therefore, suggests that therapies to target sclerostin might also improve healing of osteolytic lesions in breast cancer patients.

## Conclusions

Runx2 and CBFβ are required for the expression of genes that mediate the ability of metastatic breast cancer cells to directly modulate both osteoclast (GM-CSF, IL-11) and osteoblast (sclerostin) function. Runx2-dependent inhibition of osteoblast differentiation, by MDA-MB-231 cells, is mediated through the Wnt antagonist, sclerostin.

## Abbreviations

BMSCs: bone marrow stromal cells; CBFβ: core binding factor beta; ChIP: chromatin immunoprecipitation; DAVID: Database for Annotation, Visualization and Integrated Discovery; DKK1: Dickkopf-related protein 1; EMSA: electrophoretic mobility shift assay; FBS: fetal bovine serum; fdr: false discovery rate; GM-CSF: granulocyte-macrophage colony-stimulating factor; IgG: immunoglobulin G; IHH: Indian hedgehog homolog; IL-8: interleukin-8; IL-11: interleukin-11; NS: non-specific; OPN: osteopontin; PCA: principal component analysis; PTHrP: parathyroid hormone-related protein; P/S: Penicillin/Streptomycin; qRT-PCR: quantitative reverse transcriptase polymerase chain reaction; RANKL: receptor activator of nuclear factor kappa-B ligand; Runx2: Runt-related transcription factor 2; siRNA: small interfering RNA; shRNA: short hairpin RNA; TGF-β: transforming growth factor beta

## Competing interests

The authors declare that they have no competing interests.

## Authors' contributions

DMV performed the experiments. LZ analyzed the array data. PS is the principal investigator and was involved in the conceptualization, experimental design, discussion of data and writing of the manuscript. All authors read and approved the final manuscript.

## Supplementary Material

Additional file 1 **An Excel table with common genes for siRunx2 and siCBFβ with ≥ 2-fold change**. Click here for file

Additional file 2 **A Word table with the secreted gene products found in DAVID analysis**. Click here for file
