# Supplementary material for: Metastatic breast cancer cells inhibit osteoblast differentiation through the Runx2/CBFβ-dependent expression of the Wnt antagonist, sclerostin
Source: Breast Cancer Res. 2011 Oct 27;13(5):R106. doi: 10.1186/bcr3048 (PMC3262219; doi:10.1186/bcr3048)
Supplement: Additional file 2 — A Word table with the secreted gene products found in DAVID analysis. [file bcr3048-S2.DOCX]

**Table S1**

DAVID analysis: Secreted

| **Symbol** | **Name** | **Fold Change** | | **Function** | **Reference** |
| --- | --- | --- | --- | --- | --- |
|  |  | **siRunx2** | **siCBFb** |  |  |
| IL11 | Interleukin 11 | -3.95 | -14.80 | Secreted by breast cancer cell and promotes osteoclast formation. | **Kang Y, et al. Cancer cell.** 2003 Jun;3(6):537-49 |
| SOST | Sclerosteosis | -5.79 | -5.75 | Negative regulator of bone growth. | Van Bezooijen RL et al. Cytokine Growth Factor Rev. 2005 Jun;16(3):319-27. |
| PLAU | Plasminogen activator urokinase | -3.00 | -2.24 | Serine protease involved in degradation of the extracellular matrix and possibly tumor cell migration and proliferation | Ustach CV, et al. Mol. Cell Biol. 2005 Jul;25(14):6279-88. |
| CSF-2 | Colony stimulating factor 2 (granulocyte-macrophage) | -3.02 | -2.55 | Cytokine that controls the production, differentiation, and function of granulocytes and macrophages. Induce osteoclastogenesis by breast cancer cells. | Park BK, et al. Nat Med. 2007 Jan;13(1):62-9. Epub 2006 Dec 10. |
| IL24 | Interleukin 24 | -2.03 | -2.36 | Antiproliferative properties on melanoma cells | Jiang H, et al. PNAS August 20, 1996 vol. 93 no. 17 9160-9165 |
| IL23A | Interleukin 23, alpha subunit p19 | -2.61 | -2.12 | Associates with IL12B to form the IL-23 interleukin.  promotes production of proinflammatory cytokines | Langowski JL, et al. *Nature* 442, 461-465 (27 July 2006) |
| IL1RN | Interleukin receptor antagonist | -2.09 | -2.07 | Inhibits the activity of IL-1 by binding to its receptor. Has no IL-1 like activity | **Steinkasserer A, et al. Genomics** 1992 Jul;13(3):654-7. |
| CST7 | Cystatin F | -3.58 | -2.92 | Glycosylated cysteine protease inhibitor. an increased expression of cystatin-like metastasis-associated protein (CMAP) mRNA is involved in liver-specific metastasis in a mouse model. | Morita M. Et al. Cancer Res., 59: 151-158, 1999. |
| C4orf26 | Chromosome 4 open reading frame 26 | -3.10 | -2.85 | Unknown. | Ota T, et al. Nat Genet. 2004 Jan;36(1):40-5. Epub 2003 Dec 21. |
| C1QTNF2 | C1q and tumor necrosis factor related protein 2 | -2.06 | -2.23 | Unknown. | Gerhard DS, et al. Genome Res. 2004 Oct;14(10B):2121-7. |
